# Preclinical Studies Identify Non-Apoptotic Low-Level Caspase-3 as Therapeutic Target in Pemphigus Vulgaris

**DOI:** 10.1371/journal.pone.0119809

**Published:** 2015-03-06

**Authors:** Camille Luyet, Katja Schulze, Beyza S. Sayar, Denise Howald, Eliane J. Müller, Arnaud Galichet

**Affiliations:** 1 Molecular Dermatology, Institute of Animal Pathology, Vetsuisse Faculty, University of Bern, Bern, Switzerland; 2 DermFocus, Vetsuisse Faculty, University of Bern, Bern, Switzerland; 3 Department of Dermatology, Inselspital, Bern University Hospital, Bern, Switzerland; NCMLS, Radboud University Nijmegen Medical Center, NETHERLANDS

## Abstract

The majority of pemphigus vulgaris (PV) patients suffer from a live-threatening loss of intercellular adhesion between keratinocytes (acantholysis). The disease is caused by auto-antibodies that bind to desmosomal cadherins desmoglein (Dsg) 3 or Dsg3 and Dsg1 in mucous membranes and skin. A currently unresolved controversy in PV is whether apoptosis is involved in the pathogenic process. The objective of this study was to perform preclinical studies to investigate apoptotic pathway activation in PV pathogenesis with the goal to assess its potential for clinical therapy. For this purpose, we investigated mouse and human skin keratinocyte cultures treated with PV antibodies (the experimental Dsg3 monospecific antibody AK23 or PV patients IgG), PV mouse models (passive transfer of AK23 or PVIgG into adult and neonatal mice) as well as PV patients’ biopsies (n=6). A combination of TUNEL assay, analyses of membrane integrity, early apoptotic markers such as cleaved poly-ADP-ribose polymerase (PARP) and the collapse of actin cytoskeleton failed to provide evidence for apoptosis in PV pathogenesis. However, the *in vitro* and *in vivo* PV models, allowing to monitor progression of lesion formation, revealed an early, transient and low-level caspase-3 activation. Pharmacological inhibition confirmed the functional implication of caspase-3 in major events in PV such as shedding of Dsg3, keratin retraction, proliferation including c-Myc induction, p38MAPK activation and acantholysis. Together, these data identify low-level caspase-3 activation downstream of disrupted Dsg3 trans- or cis-adhesion as a major event in PV pathogenesis that is non-synonymous with apoptosis and represents, unlike apoptotic components, a promising target for clinical therapy. At a broader level, these results posit that an impairment of adhesive functions in concert with low-level, non-lethal caspase-3 activation can evoke profound cellular changes which may be of relevance for other diseases including cancer.

## Introduction

Pemphigus vulgaris (PV) is a severe autoimmune blistering disease affecting the epidermis, hair follicles and mucous membranes [1,2,3]. It characteristically manifests as loss of intercellular adhesion (acantholysis) between basal and suprabasal keratinocytes, where desmoglein 3 (Dsg3), the major antigenic target in PV, is most abundantly expressed [4,5]. Dsg1 can compensate for loss of Dsg3 function in the epidermis [4]; accordingly, in PV patients and mouse models, Dsg3 antibodies alone predominantly induce clinical blisters in hair follicles and mucous membranes whereas combined Dsg3 and Dsg1 antibodies concomitantly evoke epidermal blisters [3,4,6,7,8].

Dsg3 and Dsg1 are desmosomal cadherins and adhesive components of desmosomes. These robust intercellular adhesion structures confer mechanical resistance to a variety of tissues including skin. Despite their robustness, desmosomes are highly dynamic and alterations in desmosomal cadherin expression and composition are pivotal during embryogenesis, tissue homeostasis and repair [9,10]. For example, in response to injury, epidermal growth factor (EGF) stimulation or UV irradiation, mechanisms such as reversion from high to low affinity adhesive states of desmosomes [11], desmosomal cadherin endocytosis [12] and proteolytic shedding implicating consecutively caspase-3 and metalloproteases [13,14] have been described. Caspase activation was long considered an exclusive hallmark of apoptosis and hence, desmosomal remodeling has often been linked to apoptotic cell death. However, according to the recommendations of cell death classification, caspase activation alone is not sufficient to evoke apoptosis [15] because caspases, as a paradox to cell death, have been involved in proliferation, differentiation and cellular remodeling of a variety of cell types [16,17,18], which is in line with delayed keratinocyte differentiation in caspase-3 mutant mouse embryos [19]. Accordingly, depending on its level of activation, caspase-3 has been proposed as a “stress intensity sensor” acting as a switch between cell survival and death [20].

In PV, Dsg3 antibody binding directly interferes with cis- or trans-adhesion between Dsg3 molecules [21,22] thereby eliciting cellular response signals which were found to be responsible for the ultimate loss of desmosome structure and function. Specifically, pathogenic signals have been involved in re-organization and endocytosis of Dsg3 as well as a change in keratinocyte fate from differentiation to proliferation as proven by application of pharmacologic inhibitors or the use of knock-out models [23,24,25,26]. Based on the initial observation of TUNEL (TdT-mediated dUTP-biotin nick end labeling)-positive cells in lesional skin of PV patients [27,28], apoptosis was also proposed to be involved in PV pathogenesis. Independent reports on caspase activation in the neonatal PV mouse model and reduced blistering after caspase-3 inhibitor treatment supported this claim [29,30]. Accordingly, “acantholysis and apoptosis” were discussed to be “inseparable in PV”, invoking a process termed “apoptolysis” where acantholysis proceeds along apoptotic pathways resulting in cell death [31,32]. Inhibition of apoptotic pathway components including FasL was therefore suggested as potential therapy for PV patients [28,30,31,32,33]. However, doubts have been cast on the involvement of apoptosis, primarily because two independent studies failed to reveal TUNEL positive cells or apoptotic cell morphology by electron microscopy in systematic surveys of PVIgG-treated cultured HaCat keratinocytes and skin explants as well as PV patients’ skin biopsies [34,35]. Furthermore, apoptotic cell death, caspase-3 activation and nuclear accumulation of cleaved PARP were either not detected or suggested to occur after loss of adhesion. This led to the conclusion that if happening, these late events are not taking part in the acantholytic process [34,36,37]. Although the two previous studies [34,35] appeared to make a strong point against apoptosis in PV, they did not resolve the conundrum that a range of caspase-3 inhibitors was able to reduce blistering in the neonatal PV mouse model.

A variety of caspase inhibitors have been developed, some of which are in Phase I/II clinical trials [38,39]. Although these inhibitors do represent a promising adjunctive treatment option or a second line intervention for PV patients, their testing in PV clinical trials are not justified unless experimental proof of concept and the mechanisms of action of caspase-3 in PV are resolved. This prompted us to specifically evaluate the involvement of apoptosis in correlation with caspase-3 activation in PV. Here we used a combination of cultured keratinocytes, established PV mouse models [6] and previously used biopsies from PV patients [23] to firstly investigate the occurrence of apoptosis and secondly, in the same PV models, caspase-3 activation and the consequences of caspase-3 inhibition in response to the Dsg3-specific pathogenic antibody AK23 [40] or purified PVIgG.

Our data corroborate that acantholysis in PV occurs without implicating apoptosis. However, an early, transient and low-level caspase-3 activation was revealed downstream of disrupted Dsg3 cis-or trans-adhesion [21,22] which, as demonstrated by the use of pharmacological pan-caspase and caspase-3 inhibitors, functionally contributes to major pathological events in the acantholytic process. Our results support that caspase-3 is activated without triggering apoptosis but instead is an important component in the early events leading to PV blistering, thus representing a promising therapeutic target in PV.

## Material and Methods

### Keratinocyte cultures and inhibitor treatment

Mouse keratinocytes were isolated form E18.5 old C57BL/6J embryos (purchased from Central Animal Facility, University of Bern), sacrificed by decapitation and cells grown in CnT-02 medium (CELLnTEC Advanced Cell Systems AG, Bern Switzerland) and characterized as described previously [23,41,42]. Primary human epidermal keratinocytes derived from foreskin were purchased from CELLnTEC and grown in CnT-57 (CELLnTEC, Bern, Switzerland). For all experiments the medium was supplemented with 1.2 mM calcium at cell confluency to induce differentiation six hours before challenging the cultures with the standard concentration of 20 μg/ml AK23 alone [40] (a kind gift of Dr. M. Amagai, University of Tokyo, Japan) (without additional exfoliative toxin), with AK23 and 1.5 mg/ml PFIgG (Dsg1 titer 58.8), 1 mg/ml PVIgG1 (containing antibody against Dsg3 [23]) or 4 mg/ml PVIgG2 (containing antibodies against both Dsg3 and Dsg1, titer Dsg1:Dsg3; 194.4:250.2). Where indicated, higher AK23 concentrations were used to demonstrate that absence of a specific response pattern is not due to an insufficient dose. Normal human IgG (nhIgG) (Sandoglobulin) or non-specific mouse IgG (mIgG) (Equitech-Bio) were used as controls. Inhibitors (for which toxicity was excluded by morphological criteria and trypan blue exclusion) were dissolved in DMSO and added at calcium switch at the following published concentrations confirmed in a dose response study (S2 Fig.): 40 μM caspase-3 inhibitor III Ac-DEVD-CMK [43], caspase-3 inhibitor II Z-DEVD-FMK [29,44] and pan-caspase inhibitor VI Z-VAD-FMK [35] (all Calbiochem); 100 μM caspase-8 inhibitor Z-IETD-FMK [45,46]; 50 μM caspase-9 inhibitor Z-LEHD-FMK [47] (all Alexis biochemicals) and 10 μM caspase-12 inhibitor Z-ATAD-FMK [48] (BioVision).

### Mice and passive transfer

Adult and neonatal mouse models were characterized previously [6,49]. Briefly, 8-week-old C57Bl/6J mice were injected with 12.5 μg/g body weight AK23/mIgG without mechanical stress and neonatal C57Bl/6J mice with 90 μg/g body weight AK23 or mIgG together with a half-pathogenic dose PFIgG or nhIgG (12mg/g), respectively, as reported [49] and subjected to mechanical stress. For inhibitor treatment, mice were IP injected with 6 μg/g per body weight caspase-3 inhibitor III (Ac-DEVD-CMK; dissolved in DMSO and diluted in PBS) 2 hours before AK23 or AK23/PF IgG. The inhibitor concentration was determined by a dose-response study in adult mice and was found to be in the range published to prevent PFIgG-induced blistering in neonatal mice [50]. Adult mice were euthanized by inhalation with isoflurane (Piramal Healthcare, Mumbai, India) followed by cervical dislocation and neonatal mice sacrificed by decapitation. Biopsies were collected and blisters evaluated as described [49] by measuring the length of the blisters relative to the full length of the entire biopsy using imageJ on micrographs. Hair follicles blisters were counted and are presented as % of total hair follicles (minimum of 250 hair follicle/group) [6].

### Human biopsies

Biopsies of skin and oral mucosa from human PV patients have been described previously [23].

### Ethics statement

Animal experiments were approved by the ethics committee, Canton Bern, Switzerland (26/08 and BE78/11). The human biopsy tissue specimens were those obtained for the procedures carried out to confirm the diagnosis of pemphigus in affected patients. These diagnostic procedures do only require an oral consent since they represent routine laboratory tests in this context. The analyses performed with the tissue specimen did NOT involve: acquisition of information about living individuals, intervention or interaction with individuals. The research did not involve individually identifiable subjects (but anonymous information). The biopsy specimens were thus obtained and analyzed following the standards and requirement of the local Institutional board and local ethical committee valid at that time.

### TUNEL assay and FACS analysis

Mouse keratinocytes and biopsies from 8-week-old C57BL/6J mice and human PV patients [6,23] were subjected to a TUNEL assay (Roche Diagnostics, Basel, Switzerland) according to the manufacturer instructions. FACS analyses were performed as described [6].

### Caspase assay, dissociation assay and immunofluorescence microscopy

Caspase-3/7 activity was measured in lysates of mouse keratinocytes seeded in a 24-wells plate at indicated time points in triplicates using the APO-One homogenous Caspase-3/7 or Caspase-Glo 3/7 kits (Promega, Madison, Wisconsin, USA).

Dissociation assay and immunofluorescence microscopy were performed as described previously [51]. Briefly, for the dissociation assay keratinocytes were seeded in 24-well plates in duplicates, cell sheets released by dispase (2.4 U/ml, Roche Diagnostics, Basel, Switzerland) after indicated time and mechanical stress was applied by pipetting the cell sheet 10x with a P1000. Cell fragments were fixed with 3% buffered formaldehyde, stained with 0.1% crystal violet (Merck) and fragments larger than 0.1 mm^2^ counted using Paint (Microsoft) on micrographs. Immunofluorescence microscopy was performed as follows: for actin re-arrangement and keratin retraction, 8 random pictures were taken for each duplicate treatment and a minimum of 1’000 cells per duplicate treatment and group were evaluated and counted with paint (Microsoft). Alexa fluor 546 phalloidin (Sigma; 1:200) was used to stain actin and antibodies were against E-cadherin (DECMA supernatant; 1:2; kind gift of Dr. R. Kemler, Max-Plack Institute, Freiburg i.Br, Germany) and keratin 14 (A22283, Invitrogen; 1:100). For active caspase-3 staining, fixation was performed with 100% pre-cooled methanol for 10 minutes at -20°C, followed by permeabilization and blocking with 2.5% normal donkey serum, 2.5% goat serum, 0.2% Triton-X 100 and 1% BSA. Cells were then incubated with active caspase-3 antibody (AF835, R&D systems; 1:400) and revealed with Alexa Fluor 488-conjugated rabbit. Signal intensity of a minimum of 1’000 cells per duplicate treatment and group was measured on micrographs using ImageJ by excluding mitotic cells.

### Protein extraction, immunoprecipitation and western blot analysis

Protein extraction of cultured keratinocytes and mouse back skin was performed with Triton X-100 as described previously [6,23]. Proteins collected from culture media were precipitated with 2.5 volumes 100% acetone overnight at -20°C, centrifuged 10 minutes at 13000 rpm, air-dried and resuspended in Laemmli buffer. For immunoprecipitation, rabbit anti-active caspase-3 antibody (559565, BD Transduction, 2μg) and non-relevant rabbit IgG (Sigma, 2μg) were bound to protein G dynabeads (Invitrogen, #100.04D) in citrate phosphate buffer pH5 (25mM citric acid, 50mM Na_2_HPO_4_, protease inhibitor cocktail (Roche)) for 1 hour at room temperature. Antibody-coated protein G dynabeads were then incubated with 1mg Triton X-100 soluble proteins and citrate phosphate buffer pH5 (final pH of the solution >6.5) for 1 hour at room temperature. All samples of one time point were processed simultaneously. Beads were washed five times with low salt solution (Tris-HCl 10mM pH 7.5, DTT 0.5mM and protease inhibitor cocktail (Roche)) and proteins eluted with Laemmli Buffer. Protein electrophoresis and western blot analyses were performed as previously described [6] using antibodies against Dsg3 (kind gift of Dr. John Stanley, University of Pennsylvania, rabbit, 1:4000), PARP (9542, Cell Signaling Technology, rabbit, 1:1000), caspase-3 (3/CPP32, BD transduction, mouse, 1:1000; for routine analyses), caspase-3 (AF-605-NA, R&D systems, goat, 1:1000; used for immunoblotting after IP to not detect contaminating epidermal mouse IgG binding to beads) and β-tubulin for normalization (Ab6046, Abcam, 1:5000).

### Statistical analysis

Results are presented as mean ± SEM. The data were analyzed using NCSS 2007 (NCSS, Kaysville, Utah, USA). Differences between the means were assessed by 1-way ANOVA followed by Mann-Whitney *U* test or directly by Mann-Whitney *U* test to compare 2 groups.

## Results

### No evidence for DNA fragmentation and loss of membrane integrity in PV pathophysiology

**Fig 1:**
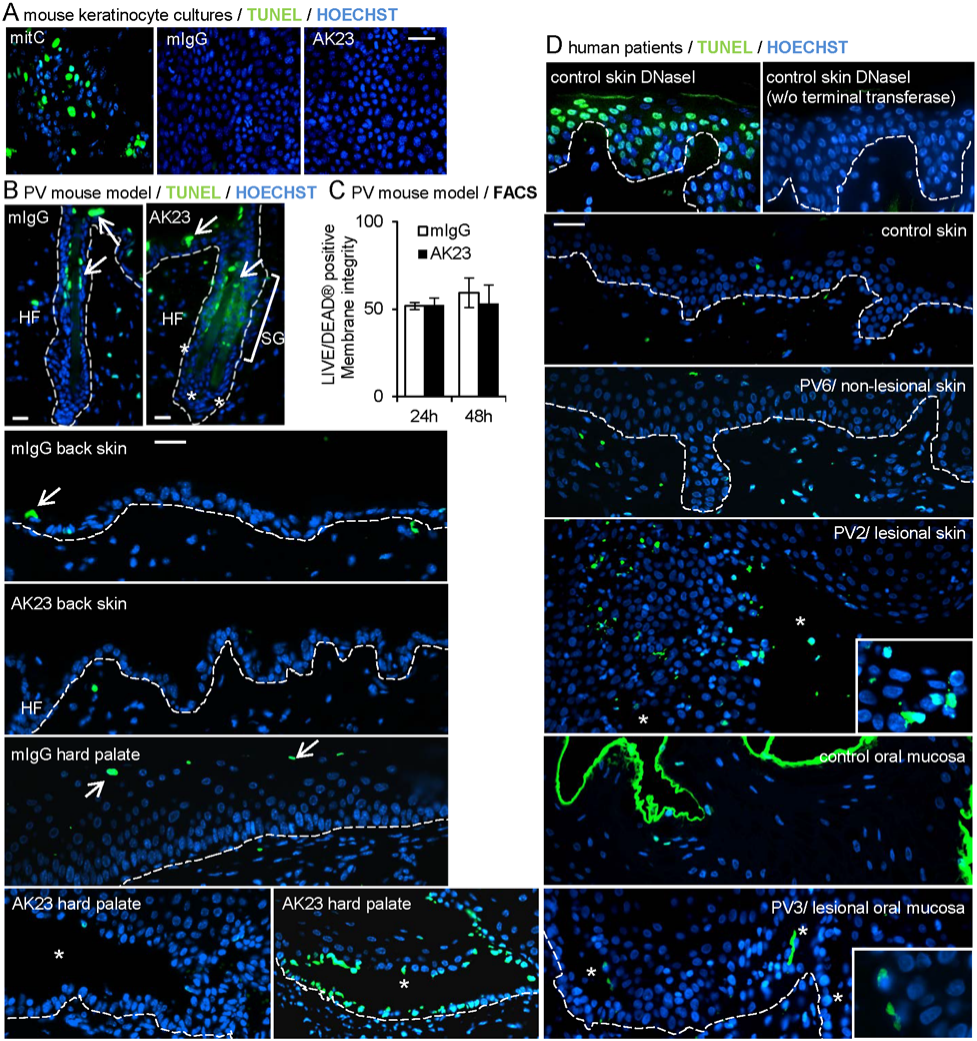
Exceptional TUNEL-positive lesions and no loss of membrane integrity in AK23-treated cultured mouse keratinocytes and 8-week-old mice or human PV patients’ biopsies. (A, B, D) Representative pictures of TUNEL staining in A, mouse keratinocyte cultures treated with 20 or 80 μg/ml AK23/mIgG for 48 hours; 20 μg/ml mitomycin C (mitC) treatment of the same culture serves as positive control for apoptosis; shown are 80 μg/ml AK23/mIgG; (n = 3 in duplicates/group and 120’000 cells/group evaluated); in B, hair follicles, back skin and hard palate of 8-week-old mice injected with 12.5 μg/g b.w. AK23/mIgG for 24h and 48h; shown are 24 hours (total mice evaluated 8; n = 2/group and time point; ≥100 hair follicles/mouse evaluated); SG: sebaceous gland, HF: hair follicle, arrows point to TUNEL positive cells in stratum corneum and sebaceous gland; in D, selected biopsies from human PV patients’ skin and oral mucosa. Note that PV patient 2 exhibits fragmentation of nuclei; DNase I-treated healthy donor skin biopsies served as positive control (high number of TUNEL positive cells) and without terminal transferase as negative control; (n = 6 PV patients, n = 2 healthy donors). Insets are twofold magnifications of selected areas. Asterisks indicate lesions. Nuclei were counterstained with Hoechst 33258. Dotted line indicates basement membrane. Bar = 75m (A), 25m (B), 125m (D). (C) Graph of LIVE/DEAD stained viable keratinocytes isolated from skin of AK23/mIgG-treated 8-week-old mice evaluated by FACS. Data are mean±SEM.

Our initial objective was to perform a systematic survey of apoptotic events in PV models and PV patients’ biopsies. Firstly, cultured mouse keratinocytes treated for 48 hours with the pathogenic antibody AK23 or mouse (m)IgG as negative control were screened for TUNEL positive cells concomitantly with loss of intercellular adhesion (S1A Fig.). As reported for human HaCat keratinocytes treated with PVIgG [35], no TUNEL positive cells were observed (evaluating roughly 120’000 cells per experiment) under our standard conditions (20 μg/ml AK23) and even when the AK23 concentration was elevated by fourfold (Fig. 1A shows 80 μg/ml). Consistent with TUNEL negativity, visual inspection did not reveal fragmented nuclei, as is expected in case of apoptosis [15]. However, on average 19% TUNEL positive cells were counted and nuclear morphology was affected when cell death was induced with high-dose mitomycin C used as positive control.

Secondly, occurrence of apoptosis was addressed in the adult PV mouse model which exhibits abundant blisters in the resting (telogen) hair follicles, microblisters in the epidermis and lesions in the palate in response to AK23 injection, a model which has so far not been addressed for apoptotic events [6]. Biopsies from these mice revealed neither TUNEL positive cells in hair follicle blisters nor in the epidermis with microblisters 24 or 48 hours after AK23 injection (Fig. 1B shows representative examples at 24 hours). Positive cells at the level of sebaceous glands and stratum granulosum, present in both control and AK23-treated biopsies, served as internal positive control. TUNEL analyses were also conducted at time points preceding (30 minutes) or at the onset of hair follicle blistering and in presence of microblisters in the epidermis (5 hours) (S1 Table). These analyses confirmed the lack of TUNEL positive cells during the process of lesion formation (data not shown). To address the occurrence of apoptosis by other means, epidermal and hair follicle keratinocytes were isolated from the back skin of these mice, stained with a commercial marker for membrane integrity and analyzed by flow cytometry. The number of viable cells was unchanged between AK23-treated and control mice (Fig. 1C). In analogy to these findings, peri-lesional, non-lesional and lesional hard palate of the same mice showed no TUNEL positive basal keratinocytes with the exception of occasional lesions which exhibited abundant TUNEL positive basal cells (Fig. 1B shows both examples).

As a third model, we investigated TUNEL positive cells in six PV patients’ biopsies previously shown to exhibit high c-Myc and increased proliferation [23]. High c-Myc has been associated with apoptosis in many cell types but not in keratinocytes which are resistant to c-Myc induced cell death [52]. In these patient’s biopsies, TUNEL positive cells were absent from non-lesional, peri-lesional and most lesional tissue (Fig. 1D shows representative examples). Rare TUNEL positive cells were present in advanced clinical lesions in skin of PV patient 2 (PV2) and suprabasal but not basal oral keratinocytes of PV patient 3 (PV3). Serving as a positive and negative control, respectively, abundant TUNEL positive cells were detected in DNaseI-treated skin and none in DNaseI-treated skin without deoxynucleotidyl transferase treatment.

In conclusion, no TUNEL positive cells or loss of membrane integrity were revealed in cultured mouse keratinocytes after loss of intercellular adhesion, in non- or peri-lesional tissue in the adult PV mouse model and in PV patient’s biopsies with high levels of c-Myc. TUNEL positive cells were also absent from most lesions positing that DNA fragmentation is not a compulsory event in PV pathogenesis and that secondary processes such as substantial mechanical stress or inflammation may induce apoptosis in keratinocytes of the palate or in advanced lesions.

### No evidence for apoptotic marker induction during PV acantholysis

**Fig 2:**
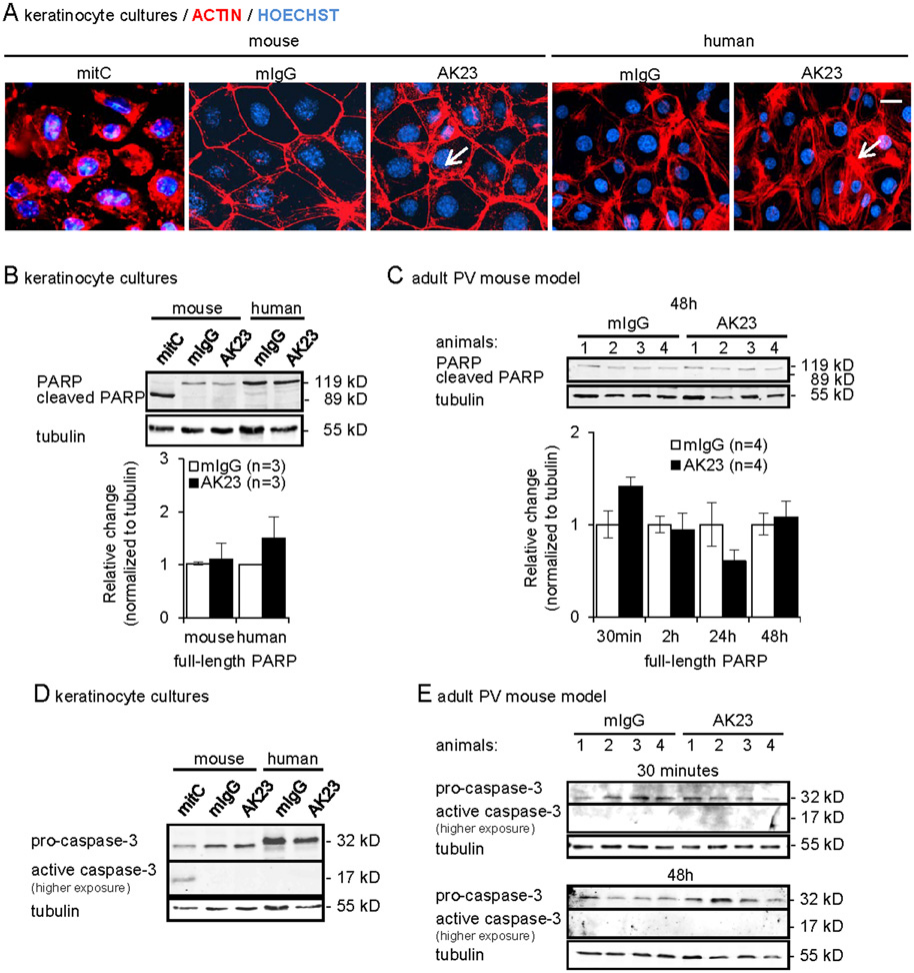
Absence of hallmarks for apoptosis in AK23-treated keratinocytes and 8-week-old mice. (A) Representative immunofluorescence micrographs for F-actin in mouse and human keratinocytes treated for 48 and 24 hours, respectively, with 20, 80 and 160 μg/ml AK23/mIgG or 20 μg/ml mitomycin C (mitC); shown are 160 μg/ml AK23/mIgG. Nuclei were counterstained with Hoechst 33258; arrows point to actin rearrangement; bar = 25μm, (n = 2/group in duplicates and 1’000 cells/group evaluated). (B-E) Representative immunoblots and graphs of Triton X-100-soluble proteins probed with indicated antibodies from B, D, mouse and human keratinocytes treated for 48 hours and 24 hours, respectively, with 20, 80 and 160 μg/ml AK23/mIgG or 20 μg/ml mitomycin C; shown are 20μg/ml (mouse cells) and 80μg/ml (human cells) AK23/mIgG; (n = 5/group); C, E, skin of 8-week-old mice injected with 12.5 μg/g b.w. AK23/mIgG for B, 30 min, 2, 24 and 48 hours and E, 30 min, 2, 5, 24 and 48 hours; shown are indicated time points; (n = 4/group). Signals were quantified, normalized to tubulin and the mean±SEM is plotted relative to mIgG set as 1.

We also addressed occurrence of apoptosis using complementary read-outs. Cytoskeletal actin is a major target for destruction during the apoptotic process [53,54]. Consistently, mitomycin C-treated mouse keratinocytes exhibited a complete collapse of the actin cytoskeleton (Fig. 2A). In contrast, subtle changes, comparable with the retraction of actin bundles from cell boarders, reported in PV during Dsg3 endocytosis [55], were observed after 48 hours in mouse keratinocytes treated with 20, 80 or 160 μg/ml AK23 (Fig. 2A shows 160 μg/ml AK23). We also investigated primary human keratinocytes treated with AK23 under conditions of loss of adhesion (Fig. 2A; S1B Fig.) as well as mouse keratinocytes incubated with PVIgG from two different patients (containing Dsg3 [23] and Dsg3/Dsg1 antibodies, respectively) (S1C Fig.). In all cases (analyzing 1’000 cells per well in duplicates), no global actin collapse comparable to mitomycin C-treated apoptotic cells was observed. Under the same conditions we also explored the steady-state levels of full-length and cleaved poly ADP-ribose polymerase (PARP), a DNA repair enzyme that is specifically inactivated through cleavage in the early course of apoptotic cell death [54]. No evidence was obtained for reduced full-length PARP (indicative for cleavage) or increased cleaved PARP in mouse and human keratinocytes treated with AK23 (Fig. 2B). As a positive control, mitomycin C treatment resulted in complete conversion of full-length PARP to cleaved/inactivated PARP. No significant decrease in full-length PARP and no cleaved PARP were further revealed in skin extracts of adult mice between 30 minutes and 48 hours after AK23 injection (Fig. 2C and upper panel shows 48 hours).

Finally we investigated activation (cleavage) of pro-caspase-3 which, among other substrates, cleaves PARP during cell death [15,54]. While mitomycin C treatment resulted in cleavage of pro-caspase-3 to the 17 kD active enzyme, the cleaved form was not detected by immunoblotting in AK23-treated mouse and human keratinocytes after 48 and 24 hours, respectively (Fig. 2D) or in mouse epidermis 30 minutes, 2, 5, 24 and 48 hours after AK23 injection (Fig. 2E shows 30 minutes and 48 hours) before, during and after detection of microlesions in the epidermis and blisters in the hair follicles (S1 Table).

In support of our initial findings, the analyses of three apoptotic markers failed to provide evidence for the occurrence of apoptotic cell death in AK23 or PVIgG-treated cultured mouse and human keratinocytes as well as epidermis and hair follicles of adult mice.

### Transient, low-level caspase-3 activation occurs in PV models

In our PV models, no evidence for a robust caspase-3 activation has been revealed, as it would be expected in case of apoptosis [15]. However, caspase inhibitors were reported to reduce blistering in neonatal mice injected with PVIgG [29,30]. Furthermore, low-level caspase-3 activity has been associated with keratinocyte terminal differentiation [19,56] but was not detected by immunoblotting applied here (Fig. 2E). This prompted us to address caspase-3 activity by more sensitive assays.

**Fig 3:**
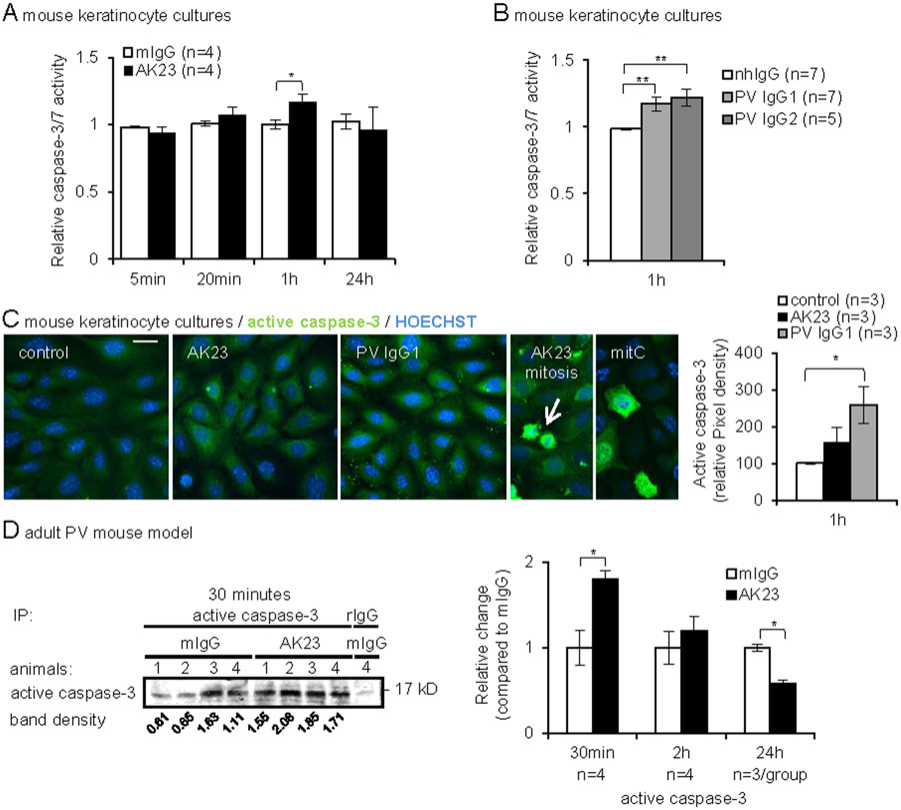
Transient, low-level caspase-3 activation in PV antibody-treated keratinocytes and 8-week-old mice. (A, B) Caspase-3/7 activity was measured in cells treated for indicated time points with A, 80 μg/ml AK23/mIgG and B, 2 mg/ml PVIgG1/nhIgG or 8 mg/ml PVIgG2/nhIgG: (n, as indicated, in triplicates). (C) Representative immunofluorescence micrographs and graph reporting active caspase-3 in mouse keratinocytes treated as in A and B for 1h or with 20 μg/ml mitomycin C (mitC) for 48h. Note that cells with mitotic figures (AK23 mitosis, arrow) have increased active caspase-3 and were therefore excluded from the quantification; fluorescence intensity was quantified by ImageJ and plotted (right panel); (n, as indicated, duplicates and 400 cells/group evaluated). (D) Active caspase-3 was immunoprecipitated (IP) from skin lysates of 8-week-old mice injected with 12.5 μg/g b.w. AK23/mIgG for 30 min, 2 and 24 hours and quantified by western blot analyses. One representative blot is shown. Specificity of the immunoprecipitation was assessed by non-relevant rabbit IgG (rIgG), shown on the right, (n/group as indicated). The mean±SEM of quantified and normalized signals is reported as relative change compared to mIgG set as 1, *p<0.05, **p<0.01.

As compared to robust caspase activation in mitomycin C-treated cells (S2A Fig.), mouse keratinocytes treated with 20 or 80 μg/ml AK23 exhibited an early transient, low-level caspase-3/7 activation with a peak at 1 hour measured by two different caspase-3/7 activity assays (the substrate is specific for caspase-3 and -7) (Fig. 3A shows results of the Caspase-Glo 3/7 assay for 80 μg/ml AK23). PVIgG from two patients (containing Dsg3 [23] and Dsg3 and Dsg1 antibodies, respectively) gave similar results (Fig. 3B). In support of these findings, semi-quantitative immunofluorescence microscopy revealed a subtle but significant increase in activated caspase-3 quantified on micrographs of mouse keratinocytes 1 hour after AK23 and PVIgG exposure (Fig. 3C; quantification of 1’000 cells per well in duplicates). In AK23- and PVIgG-treated cells, active caspase-3 mainly localized to the cytoplasm while the association with DNA in mitotic cells served as a positive control in agreement with a role of caspase-3 in mitotic spindle checkpoint control [57]. Furthermore, high levels of active caspase-3 were detected in the cytoplasm and nucleus of mitomycin C-treated apoptotic cells.

The findings obtained in cultured keratinocytes prompted us to address potential low-level caspase-3 activation also in AK23-treated and control adult mice. Lysates from mouse skin must be harvested with proteinase inhibitors to prevent protein degradation, precluding the testing by caspase activity assays. To reveal low-level caspase-3, lysates were subjected to immunoprecipitation using activated caspase-3 antibodies. Consistent with the involvement of caspase-3 in keratinocyte differentiation [19,56], activated caspase-3 was detected in control skin and was transiently increased 30 minutes after AK23 injection, before the first appearance of hair follicle and oral blisters (S1 Table), as revealed in a time course experiment (Fig. 3D), that is.

### Caspase-3 functionally contributes to loss of intercellular adhesion in PV models

**Fig 4:**
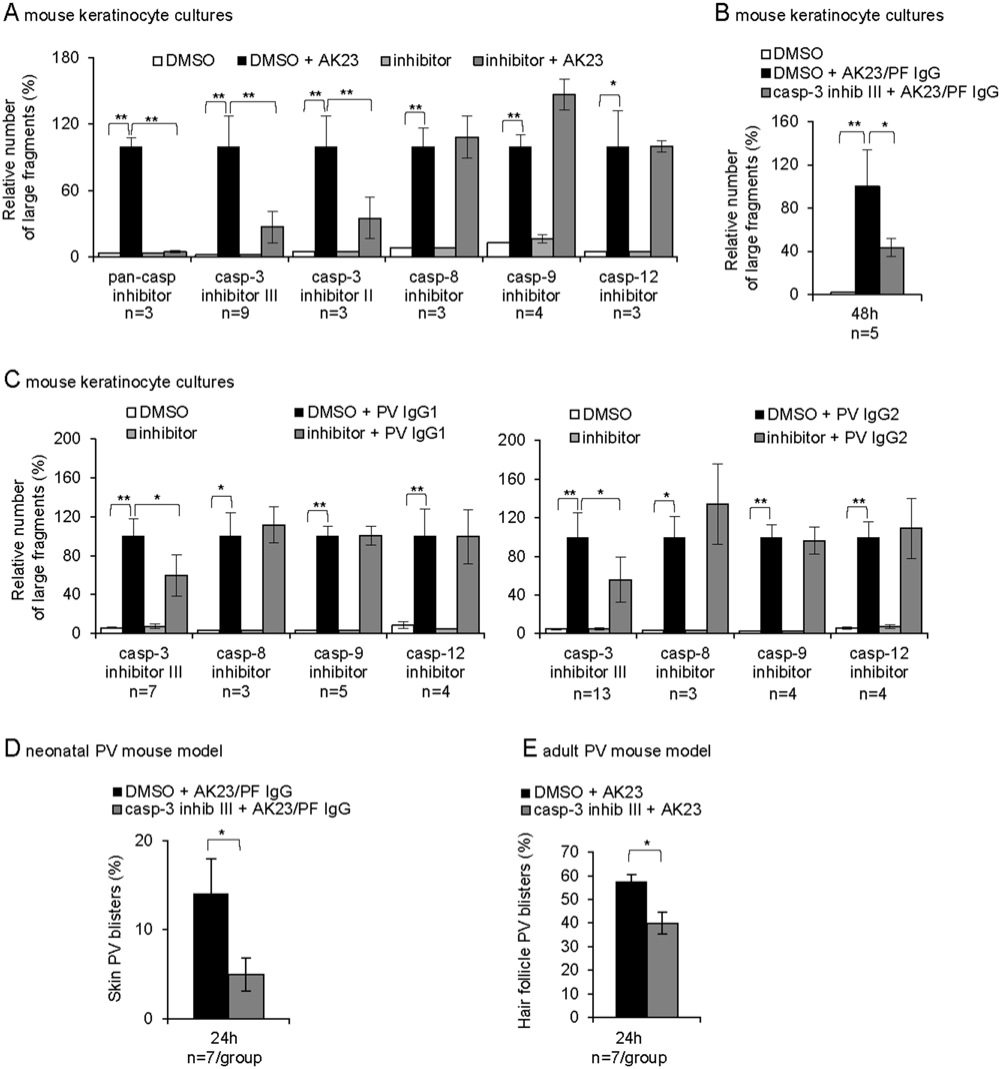
Involvement of low-level caspase-3 in loss of intercellular adhesion *in vitro* and *in vivo*. (A-C) Dissociation assay; mouse keratinocyte treated with A, 20 μg/ml AK23/mIgG; B, 20 μg/ml AK23/mIgG with 1.5mg/ml PFIgG/nhIgG or C, 1mg/ml PVIgG1/nhIgG or 4mg/ml PVIgG2/nhIgG with or without the indicated inhibitors. The number of generated fragments is presented as mean±SEM relative to DMSO/pathogenic antibody treatment set as 100%; (n, as indicated, in duplicates). (D-E) Percentage of D, PV skin blisters in AK23/PF IgG-injected neonatal mice and E, hair follicle PV blisters in AK23-injected 8-week-old mice, with or without caspase-3 inhibitor III. (n, as indicated). Data are mean±SEM, *p<0.05, **p<0.01.

To address whether the low-level caspase-3 activation observed prior to loss of intercellular adhesion functionally contributes to acantholysis, we used caspase inhibitors with different specificities and targets. A dissociation assay [51] revealed that AK23-mediated loss of intercellular adhesion was significantly reduced in cultured mouse keratinocytes pre-treated with a pan-caspase and two caspase-3 inhibitors (Z-VAD-FMK, Z-DEVD-FMK (II), Ac-DEVD-CMK (III); S2B and S2C Figs. for efficiency control) (Fig. 4A). The pan-caspase inhibitor, which also targets other proteases such as calpain [58], had the strongest effect. We therefore continued to use caspase-3 inhibitor III with high specificity for caspase-3 [59]. Similar results than in mouse keratinocytes treated with AK23 were obtained for human keratinocytes (S3A Fig.) as well as for AK23/PFIgG (combined Dsg3/Dsg1 antibodies), PVIgG1- and PVIgG2-incubated mouse keratinocytes in presence of the caspase-3 inhibitor III (Fig. 4B and 4C).

Known activators of caspase-3 in the classical apoptotic pathway are either caspase-8, downstream of the death receptor, or caspase-9/-12, following mitochondrial/endoplasmic reticulum stress [54]. Caspase-8, -9 and -12 inhibitor pre-treatment, at concentrations tested to abrogate mitomycin C-induced caspase-3 activation (S2A and S2D–2F Fig.), failed to prevent AK23- and PVIgG-induced loss of cell-cell adhesion in mouse and human keratinocytes (Fig. 4A and 4C; S3B and S3C Fig.). Without excluding some low-level activation, this suggested that these caspases are not implicated in the process leading to caspase-3-mediated loss of intercellular adhesion in these cells and that other activators than classical apoptotic pathway components are responsible to induce low-level caspase-3.

*In vivo*, in PVIgG-treated neonatal mice, pan-caspase and caspase-3 inhibitors were shown to prevent skin blistering [29,30]. To confirm these results, we pre-treated neonatal mice with the caspase-3 inhibitor III two hours prior to injecting AK23/PFIgG to induce clinical epidermal blisters as established previously [6,49]. As measured over the entire epidermis [49], inhibitor pre-treatment, at the dose preventing blistering in a mouse model for pemphigus foliaceus [50], reduced epidermal lesions on average by 60% (Fig. 4D). Like adult mice (Fig. 1B and 1C), these AK23/PFIgG-injected neonatal mice also lacked TUNEL positivity in basal epidermal keratinocytes (data not shown). Pre-treatment of adult mice with the same relative inhibitor dose per body weight also significantly reduced hair follicle blistering assessed at 24 hours (Fig. 4E).

In summary, caspase-3 but not caspase-8, -9 and -12 inhibitor pre-treatment revealed that the transient and low-level caspase-3 activation, shown here to occur prior to loss of adhesion *in vitro* and *in vivo*, is implicated in PV acantholysis without involving apoptotic cell death pathways (Figs. 1, 2 and 4C).

### Involvement of transient, low-level caspase-3 in crucial events leading to acantholysis

**Fig 5:**
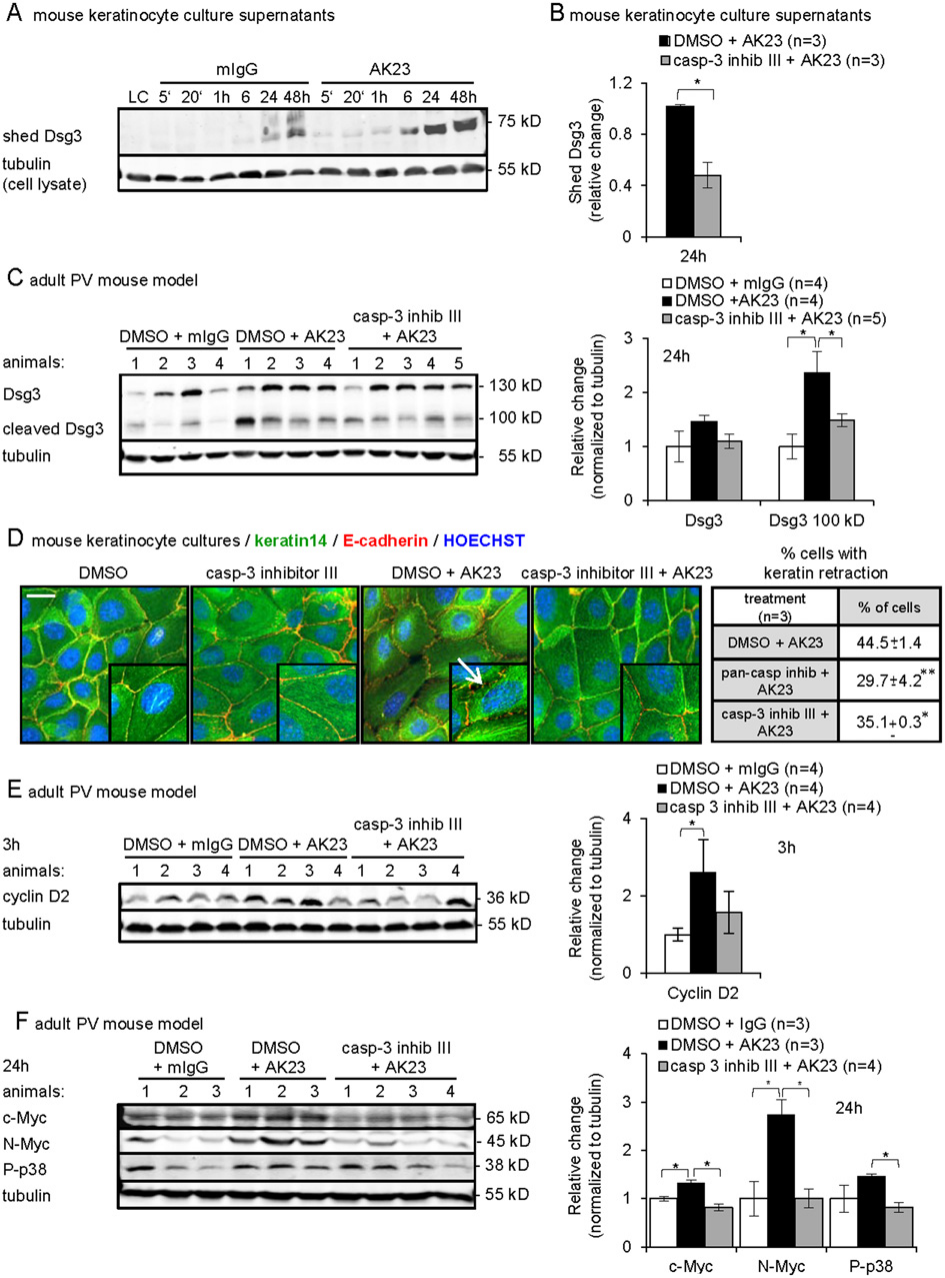
Functional contribution of low-level caspase-3 to the acantholytic process *in vitro* and *in vivo*. (A-C and E-F) Representative immunoblots and graphs for indicated proteins of A-B, culture medium of mouse keratinocytes treated with A, 20 or 80 μg/ml AK23/mIgG for the indicated time (shown are 20 μg/ml, n = 3) and B, 20 μg/ml AK23 with or without caspase-3 inhibitor for 24 hours (n = 3/group in triplicate); the mean±SEM of quantified and normalized signals for shed Dsg3 is reported as relative change compared to DMSO/AK23 set as 1, *p<0.05; (C and E-F) Triton X-100-soluble protein fractions from skin of AK23/mIgG-injected 8-week-old mice with or without caspase-3 inhibitor III treatment for the indicated time (n/group, as indicated); signals were quantified, normalized to tubulin and the mean±SEM is plotted relative to DMSO/mIgG set as 1, *p<0.05. (D) Percentage±SEM and representative immunofluorescence micrographs of mouse keratinocytes with keratin retraction and cell detachment after treatment for 48h with 80 μg/ml AK23/mIgG with or without pan-caspase and caspase-3 inhibitor III, (n = 3, 1’000 cells/group evaluated). Insets are 1.5 fold magnification of selected areas; arrow points to keratin retraction; nuclei were counterstained with Hoechst 33258; bar = 10 μm.

We further tested the functional contribution of caspase-3 to key events in loss of intercellular adhesion. Remodeling of desmosomes is a major feature in PV and involves for instance Dsg3 shedding as observed in PVIgG-treated human keratinocytes [44]. Desmosomal cadherins can be processed by caspase-3 followed by metalloprotease cleavage, providing a 100 kD membrane anchored and a 75 kD shed N-terminal Dsg3 fragment, respectively [13,14]. We hypothesized that Dsg3 shedding in PV might involve caspase-3. To address this possibility, equal volumes of culture supernatants from AK23/mIgG-treated mouse keratinocytes were analyzed by immunobloting. Twenty or 80 μg/ml AK23 treatment resulted in enhanced shedding of a 75 kD Dsg3 extracellular fragment into the supernatant as compared to differentiating control cells, which also exhibited some Dsg3 cleavage consistent with caspase-3 activation during differentiation [19,56] (Fig. 5A shows 20 μg/ml). The caspase-3 inhibitor III significantly reduced the AK23-mediated Dsg3 shedding (Fig. 5B).

In Triton X-100 soluble epidermal protein extracts from AK23 treated mice, the 75 kD shed Dsg3 extracellular fragment was not detectable, probably due to rapid clearance *in vivo* (data not shown). However, the membrane anchored, 100 kD cleaved Dsg3 fragment, spanning the Dsg3 extracellular, transmembrane and partial intracellular domain [14], was increased 24 hours after AK23-injection and pre-treatment with the caspase-3 inhibitor significantly reduced AK23-induced Dsg3 cleavage (Fig. 5C).

Keratin retraction form cell boarders is a predominant feature accompanying loss of intercellular adhesion and is readily observed in cultured mouse keratinocytes treated with PVIgG [26]. The immunofluorescence-aided evaluation of keratinocytes treated with AK23 for 48 hours (1’000 cells/group in duplicate of three experiments) revealed on average 44% cells with retracted keratin filaments from cell boarders (Fig. 5D). A six hours pre-incubation with the pan-caspase inhibitor or caspase-3 inhibitor III prior to addition of AK23 significantly reduced this phenomenon.

Based on the functional implication of low-level caspase-3 in Hela and T-cells proliferation [57,60], we reasoned that the early low-level caspase-3 in PV could affect both early and late proliferation markers reported to be upregulated in the adult PV model [6]. Triton-X soluble lysates from AK23-injected adult mice revealed that pre-treatment with the caspase-3 inhibitor III dampened the early increase in cyclin D2, as measured after 3 hours, and also significantly reduced c-Myc and N-Myc as well as p38MAPK, another player in PV, measured after 24 hours [37] (Fig. 5E and 5F).

In summary, our data reveal that transient, low-level caspase-3 is activated outside of classical apoptotic circuits and contributes to major events in PV acantholysis *in vitro* and *in vivo*.

## Discussion

Our results revealed a transient, low-level caspase-3 activation downstream of disrupted Dsg3 cis- or trans-adhesion [21,22], which occurs early in PV acantholysis, is uncoupled from apoptosis or classical apoptotic pathways and, as judged from caspase-3 inhibitor treatments, contributes to major events in PV pathogenesis; these are increased expression of proliferation markers including Myc family members, p38MAPK activation, cleavage of Dsg3, keratin retraction as well as loss of intercellular adhesion. As a primary outcome, these preclinical studies provide the basis to justify Phase I clinical trials for PV patients involving caspase-3 inhibitors and, secondly, may resolve the conundrum of apoptosis in PV.

Initial evidence in support of apoptosis in PV was based on two reports of TUNEL-positive cells in lesions of PV patients [27,28]. Loss of intercellular adhesion or acantholysis in PV undeniably evokes an aspect of apoptosis, which is the reduction of the cellular volume termed cell “shrinkage” [31]. However, as recommended by the Nomenclature Committee on Cell Death [15], other morphological criteria, in particular chromatin condensation and nuclear fragmentation have to occur to confirm apoptosis. A recent survey of four PV patient’s biopsies using electron microscopy [34] as well as our analyses by Hoechst 33258 staining in cultured keratincoytes, PV mouse models and PV patient’s biopsies (Fig. 1) now provide strong evidence that these typical morphological signs of apoptosis are not commonly seen in PV. Furthermore, the screen of all currently available experimental PV models as well as patients biopsies for TUNEL positive cells in concert with membrane and cellular integrity or apoptosis-related enzymological changes, done here and in part also in two other studies [34,35], did also not hint at a consistent cellular change in progression to the requested “point-of-no-return” featuring cell death. The occasional rather than systematic occurrence of TUNEL positive PV lesions, observed here and in previous studies [27,28], suggests that secondary conditions, which are unrelated to the mechanism of lesion formation *sensu strictu* may favor cell death in acantholytic cells. These may involve extensive mechanical stress through scratching or secondary infections. Together with the two other studies [34,35] our results obtained in cultured keratinocytes, mouse models and human patients biopsies posit that apoptosis is not a mandatory event in the progression to clinical lesions in PV.

Besides TUNEL positivity, apoptosis-unrelated effectors such as activated EGFR and c-Myc have also been proposed as markers of apoptosis in PV [32,61,62]. Incidentally, however, EFGR represents a survival factor in case of loss of adhesion in keratinocytes [63] and these cells are resistant to the c-Myc-induced apoptosis seen in other cell types [52].

The major arguments brought forward in support of apoptosis or activation of apoptotic pathways in PV was, however, the direct and in particular indirect evidence (by use of caspase inhibitors preventing loss of adhesion) for caspase activation [27,28,29,30,46,64]. Nevertheless, caspase-3 activation was not consistently detected in all studies [34,35]. These seemingly controversial data can now be explained by the findings described in this study. Our data confirm absence of robust caspase-3 activation before, during and after blister formation *in vivo* (Fig. 2E, S1 Table). They instead revealed low-level caspase-3 activation, which occurs in a transient manner *in vitro* and *in vivo* and functionally contributes to PV pathogenesis as demonstrated by pharmacological caspase-3 inhibition ameliorating blistering. By revealing low-level caspase-3 activation in the same models which failed to exhibit apoptotic pathway activation, these results now allow us to propose a paradigm shift; pathological caspase-3 activation occurs in PV but is of low-level and uncoupled from apoptosis and classical apoptotic pathways involving initiator caspases-8, -9 and -12 (as blocking the initiator caspase-8, -9 and -12 did not prevent loss of intercellular adhesion).

A role of caspases independent of apoptosis has been appreciated for many years [16]. As a paradox to apoptosis, caspase-3 has in particular been implicated in the regulation of cell proliferation, migration and differentiation when activated in a transient manner and at low-levels [16,17,18,60,65]. For example, caspase-3 but not caspases-1, -6, -7, -8, -9, or -10 was found to be required “periodically” in pre-mitotic HeLa cells without inducing apoptosis [57]. Reminiscent of our own findings, the authors of the study on HeLa cells further discussed that the balance between pro- and anti-apoptotic functions relies on the intensity (high versus low) and duration of caspase-3 activation. In support of their claims, transient low-level caspase-3 activation in early antigen-driven CD8^+^ T cell activation correlated with proliferation but not with cell death [60]. Consistently, in PV mouse models and patients epidermis, enhanced proliferation in concert with increased c-Myc and cyclin D1 have been observed [6,23] and a role of caspase-3 in supporting proliferation in PV is suggested here by the reduction of some of these pro-proliferative mediators in response to caspase-3 inhibitor treatment.

In T cells with non-apoptotic caspase-3 activation, this enzyme was found to primarily associate with the plasma membrane [65]. Based on the knowledge that executioner caspases such as caspase-3 must enter the nucleus to trigger apoptosis, this conveys that the subcellular localization of caspase-3 may also impact on its apoptotic activity. A non-apoptotic function of caspase-3 at the plasma membrane, as suggested by our results on Dsg3 shedding, may involve the cleavage of molecules implicated in intercellular adhesion and remodeling of desmosomes. Desmosomal cadherins in addition to the plaque proteins plakoglobin, plakophilin and desmoplakin were indeed found to represent intracellular substrates for caspase-3 during desmosome remodeling [14,66,67]. Furthermore, caspase-3-cleaved Dsg3 serves as a substrate for extracellular metalloproteases resulting in shedding of Dsg3 from the surface [14]. Shed Dsg3, as observed here in PV but to some extent also in differentiating keratinocytes, might interfere through competition with transadhering Dsg3 to enhance loss of adhesion in PV or remodel adhesion during keratinocyte differentiation, respectively.

With regards to potential pathways activating low-level, non-apoptotic caspase-3, TRAIL-induced TNF receptor activation was found to have this ability in the context of normal human keratinocytes differentiation further implicating p38MAPK [56]. Remarkably, p38MAPK activation has been involved in PV pathogenesis [68], and its decrease by caspase-3 inhibition, shown here in the PV mouse model, stipulates that p38MAPK could, like cyclins, c-Myc and N-Myc, be dependent on caspase-3 activation in PV. Another candidate which may potentially cooperate with and in particular induce caspase-3 activation in keratinocytes under stress is EGFR, which is activated in our PV mouse models [6,49]. In mouse and human, caspase-3 can be directly activated through effectors such as granzyme B [69,70] which depends on EGFR signaling as described in sub-lethally UV-B irradiated keratinocytes [71].

A particularly interesting role of caspase-3 in cell survival rather than cell death has recently been described in response to mild cellular stress [20]. Low-level caspase-3 differentially cleaves p120 RasGAP resulting in activation of the survival pathway Akt and an anti-apoptotic feedback loop preventing robust caspase-3 activation. The initiation of an anti-apoptotic stress response upon antibody binding to Dsg3 may be supported by the increase in heat shock protein 27 (hsp27) downstream of p38MAPK, which was described in human PV patients skin and cultured keratinocytes treated with PVIgG or cloned PV patients’ antibodies [24,72]. Hsp27 is mostly anti-apoptotic; it maintains redox homeostasis, mitochondria stability and can block cytochrome c release. At the same time it prevents robust caspase-3 activation and initiates survival pathways such as Akt to repair cellular damage [73]. As discussed above, a potential interdependence between caspase-3 and p38MAPK is suggested in our study by caspase-3 inhibitor treatment. This interdependence is also compatible with their time frame of activation; both effectors are both activated early after disruption of Dsg3 adhesion. Furthermore, p38MAPK inhibitor-treated mice injected with PVIgG [68] as well as caspase-3 inhibitor treatment, shown here, prevent key pathological events and ameliorate blistering. It is therefore conceivable that subsequent to disrupted Dsg3 trans- or cis-adhesion and pathological signal activation involving caspase-3, a cellular response aiming at cell survival and repair is mounted in PV. Cell survival and repair, as opposed to apoptosis and clearance of damaged cells is supported by the hair follicle phenotype of Dsg3-/- mice, in which blisters in the resting hair follicle resolve allowing for hair regrowth in anagen [74]. Similarly, in the 8-week-old mice injected with AK23 used here, hair follicle lesions are repaired without exhibiting apoptosis and cell-cell contact is re-established prior to the next anagen entry (Schulze et al, unpublished). Potential repair mechanisms in PV have so far not been addressed and warrant further investigation.

A particularly interesting study was published during the revision of this manuscript. Using atomic force microscopy, the authors showed that pathogenic single-chain PV antibodies irreversibly alter the stiffness of HaCaT cells implicating caspases (as shown by a pan-caspase inhibitor) but not FasL [75]. Although caspase activation was addressed as an apoptotic pathway mediator, both our studies are in agreement that apoptotic cell death is not involved in PV pathogenesis.

In conclusion, our results support a new model in which PV antibody binding triggers a cellular signaling response implicating early transient, low-level caspase-3 activation. This activity is uncoupled from apoptosis and engages a variety of cellular processes preceding acantholysis such as remodeling of Dsg3 adhesion via proteolytic cleavage and the reorganization of the keratin network. This scenario is ideally suited to propose caspase-3 as an adjunctive target to reduce blistering in PV.

## Supporting Information

S1 Fig Dissociation assays in AK23-treated mouse and human keratinocytes and actin remodeling in PVIgG-treated mouse keratinocytes. (A-B) Dissociation assays: Graphs depict the number of fragments larger than 0.1 mm^2^ generated after AK23 treatment and application of mechanical stress of A, C57BL/6J mouse keratinocytes at 48 hours, (n = 3/group in duplicates); note that 20 μg/ml AK23 treatment for 48 hours was chosen as standard condition as illustrated in the right panel; B, primary human foreskin keratinocytes, with 80 μg/ml AK23 for 24 hours set as standard. To not perturb AK23-induced signaling pathways, human keratinocytes were incubated without exfolative toxin (which cleaves compensatory Dsg1 [4]. Data are presented as mean±SEM, (n = 3 in duplicates) **p< 0.01. (C) Representative immunofluorescence micrographs for F-actin in mouse keratinocytes treated with 1 mg/ml PVIgG1/nhIgG or 4 mg/ml PVIgG2/nhIgG for 24h; nuclei were counterstained with Hoechst 33258, bar = 25m, (n = 1 in duplicates).
(TIFF)

S2 Fig Caspase inhibitor titrations. (A) Caspase-3/7 activity measured by Caspase-Glo 3/7 assay in mouse keratinocytes treated for 24 hours with 20 μg/ml mitomycin C with or without 40μM caspase-3 inhibitor III, 100μM caspase-8 inhibitor, 50μM caspase-9 inhibitor or 10μM caspase-12 inhibitor. Data are presented as mean±range relative to DMSO set as 1, n = 11, 11, 9, 2, 2 and 2, respectively, in triplicates, *p<0.05. Note that caspase-3, -8, -9, -12 inhibitors prevent caspase-3 activation in mitomycin treated cells. (B-F) Dose response for caspase inhibitors: dissociation assay; mouse keratinocytes treated for 48 hours with 20 μg/ml AK23 with or without indicated caspase inhibitors in the range of concentrations previously reported: (B) caspase-3 inhibitor III (Ac-DEVD-CMK) [1], (C) caspase-3 inhibitor II (Z-DEVD-FMK) [2,3], (D) caspase-8 inhibitor (Z-IETD-FMK) [4,5], (E) capase-9 inhibitor (Z-LEHD-FMK) [6] and (F) caspase-12 inhibitor (Z-ATAD-FMK) [7]. The concentrations selected for analysis (Fig. 4) are in bold. The number of generated fragments is presented as mean±SEM or ±range relative to DMSO/AK23 treatment set as 100%; (n = as indicated in duplicates), *p<0.05, **p<0.01.
(TIF)

S3 Fig Caspase-3 is involved in AK23- and PVIgG-mediated loss of intercellular adhesion in primary human keratinocytes. (A-C) Dissociation assays; graphs depict the number of fragments generated after treatment for 24 hours with 80 μg/ml AK23/mIgG, 1 mg/ml PVIgG1/nhIgG or 4 mg/ml PVIgG2/nhIgG with or without indicated inhibitors (concentrations described in Material and Methods). Data are presented as mean±SEM relative to AK23 or PVIgG treatment set as 100%, (n = 3/group done in duplicates); *p< 0.05, **p < 0.01.
(TIF)

S1 Table Blister quantification on H&E sections. Hair follicle (~100 hair follicles evaluated per animal and time point) and tissue blisters were counted on consecutive sections. The number of affected over total animals tested per blister site and time point after AK23 injection is indicated.
(TIF)
