# Supplementary figures and images for: Preclinical Studies Identify Non-Apoptotic Low-Level Caspase-3 as Therapeutic Target in Pemphigus Vulgaris
Source: PLoS One. 2015 Mar 6;10(3):e0119809. doi: 10.1371/journal.pone.0119809 (PMC4352034; doi:10.1371/journal.pone.0119809)

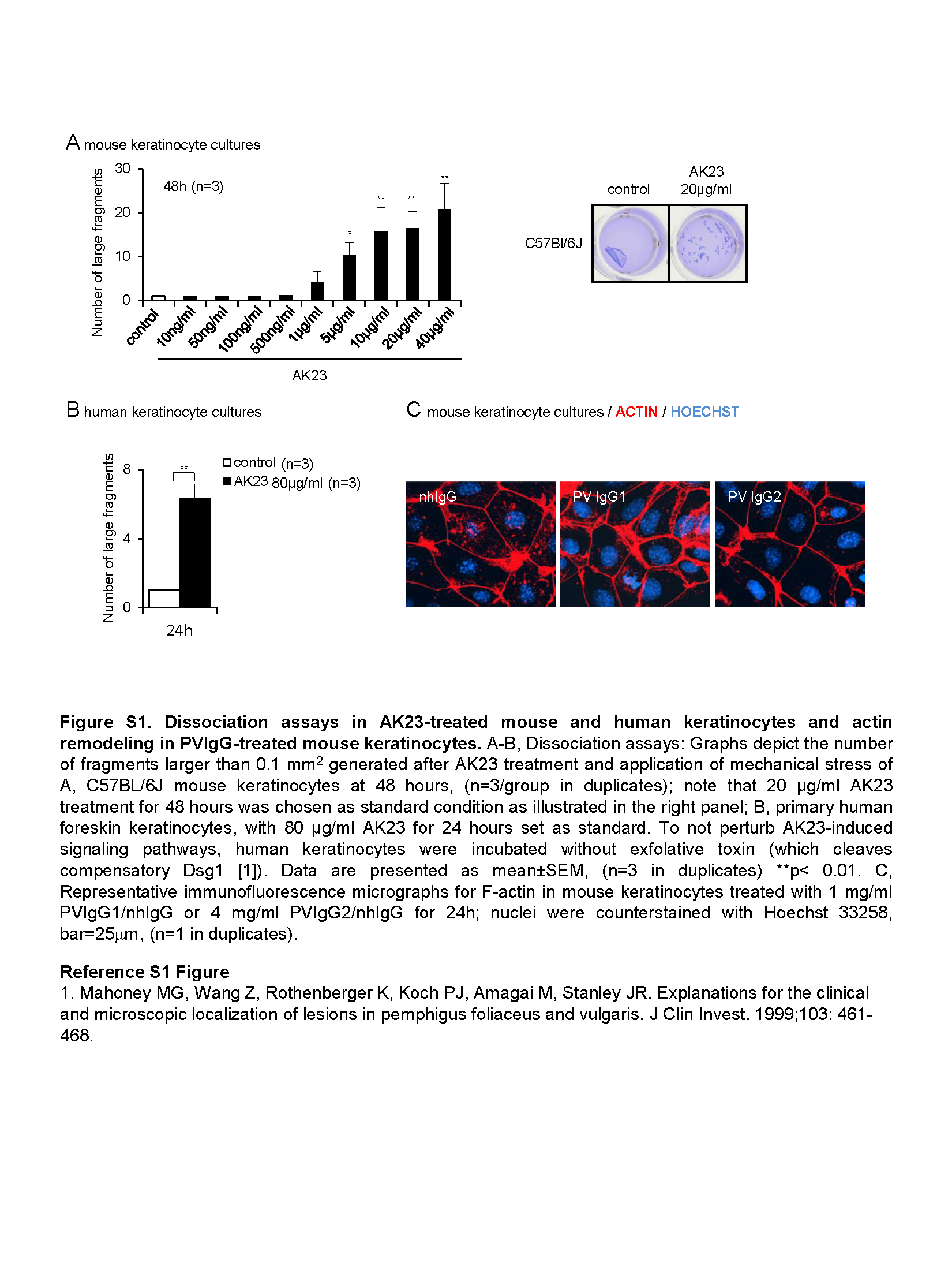

Supplement: S1 Fig — (A-B) Dissociation assays: Graphs depict the number of fragments larger than 0.1 mm2 generated after AK23 treatment and application of mechanical stress of A, C57BL/6J mouse keratinocytes at 48 hours, (n = 3/group in duplicates); note that 20 μg/ml AK23 treatment for 48 hours was chosen as standard condition as illustrated in the right panel; B, primary human foreskin keratinocytes, with 80 μg/ml AK23 for 24 hours set as standard. To not perturb AK23-induced signaling pathways, human keratinocytes were incubated without exfolative toxin (which cleaves compensatory Dsg1 [4]. Data are presented as mean±SEM, (n = 3 in duplicates) **p< 0.01. (C) Representative immunofluorescence micrographs for F-actin in mouse keratinocytes treated with 1 mg/ml PVIgG1/nhIgG or 4 mg/ml PVIgG2/nhIgG for 24h; nuclei were counterstained with Hoechst 33258, bar = 25m, (n = 1 in duplicates). (TIFF) [file pone.0119809.s001.tiff]

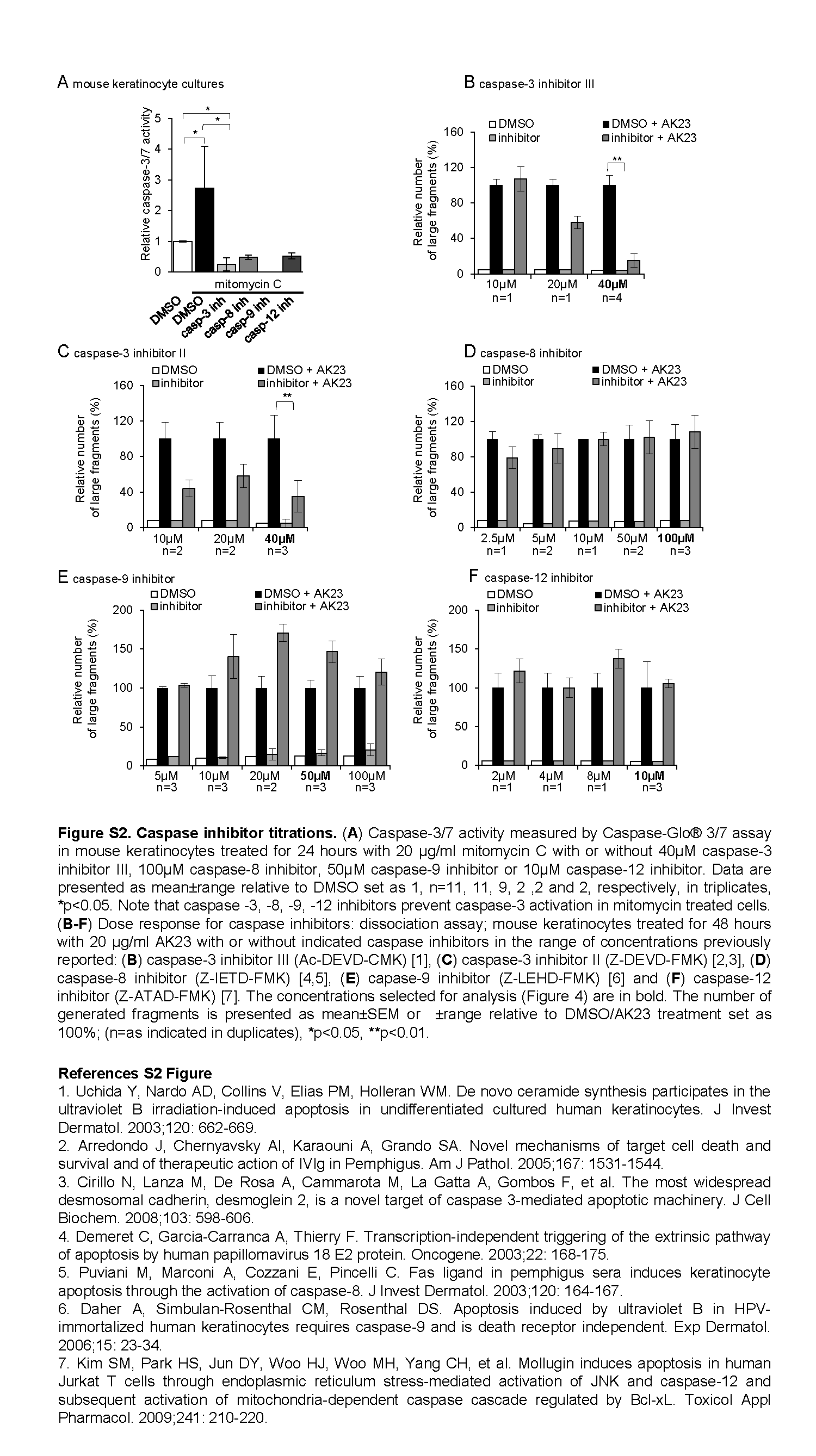

Supplement: S2 Fig — (A) Caspase-3/7 activity measured by Caspase-Glo 3/7 assay in mouse keratinocytes treated for 24 hours with 20 μg/ml mitomycin C with or without 40μM caspase-3 inhibitor III, 100μM caspase-8 inhibitor, 50μM caspase-9 inhibitor or 10μM caspase-12 inhibitor. Data are presented as mean±range relative to DMSO set as 1, n = 11, 11, 9, 2, 2 and 2, respectively, in triplicates, *p<0.05. Note that caspase-3, -8, -9, -12 inhibitors prevent caspase-3 activation in mitomycin treated cells. (B-F) Dose response for caspase inhibitors: dissociation assay; mouse keratinocytes treated for 48 hours with 20 μg/ml AK23 with or without indicated caspase inhibitors in the range of concentrations previously reported: (B) caspase-3 inhibitor III (Ac-DEVD-CMK) [1], (C) caspase-3 inhibitor II (Z-DEVD-FMK) [2,3], (D) caspase-8 inhibitor (Z-IETD-FMK) [4,5], (E) capase-9 inhibitor (Z-LEHD-FMK) [6] and (F) caspase-12 inhibitor (Z-ATAD-FMK) [7]. The concentrations selected for analysis (Fig. 4) are in bold. The number of generated fragments is presented as mean±SEM or ±range relative to DMSO/AK23 treatment set as 100%; (n = as indicated in duplicates), *p<0.05, **p<0.01. (TIF) [file pone.0119809.s002.tif]

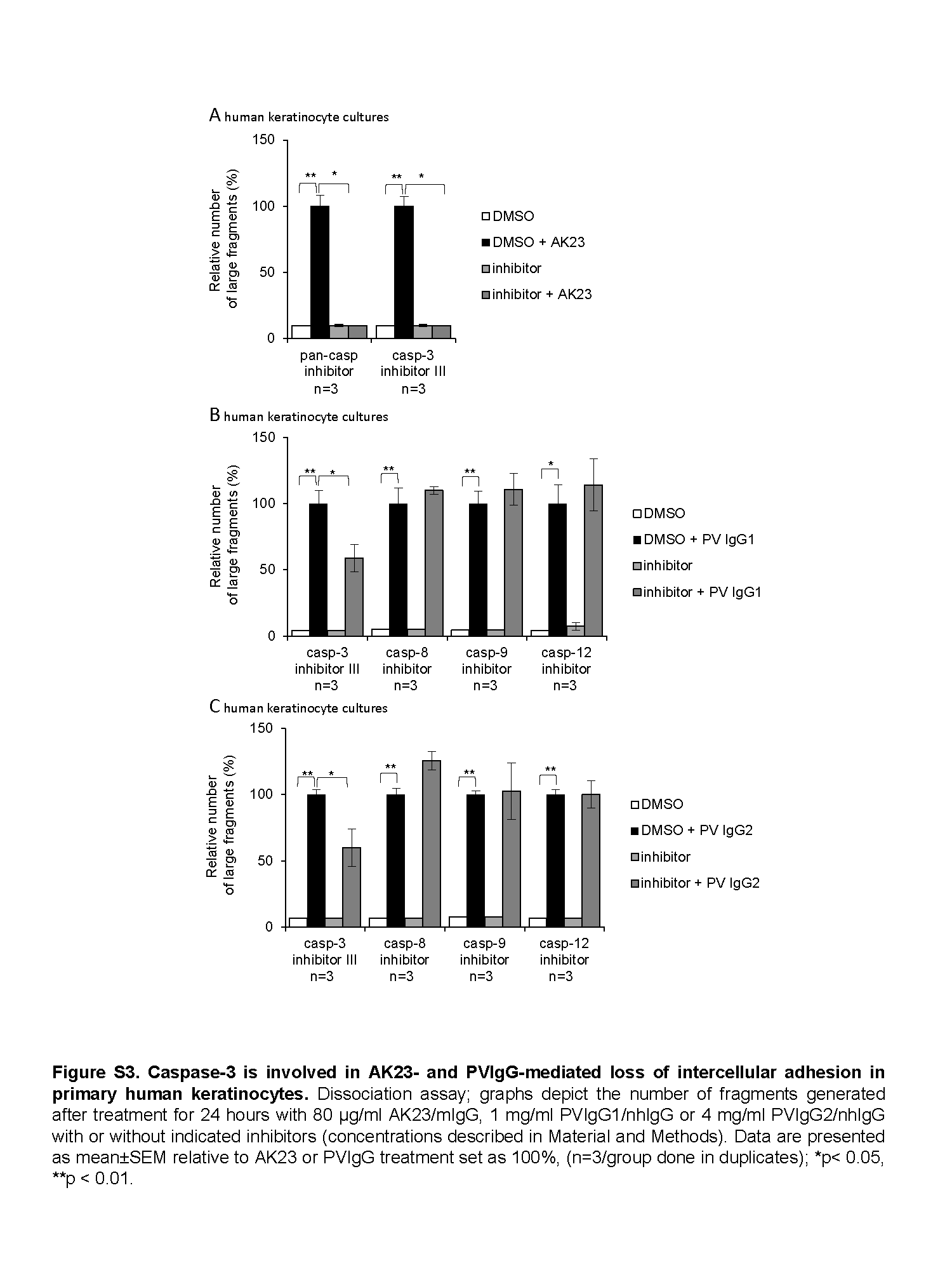

Supplement: S3 Fig — (A-C) Dissociation assays; graphs depict the number of fragments generated after treatment for 24 hours with 80 μg/ml AK23/mIgG, 1 mg/ml PVIgG1/nhIgG or 4 mg/ml PVIgG2/nhIgG with or without indicated inhibitors (concentrations described in Material and Methods). Data are presented as mean±SEM relative to AK23 or PVIgG treatment set as 100%, (n = 3/group done in duplicates); *p< 0.05, **p < 0.01. (TIF) [file pone.0119809.s003.tif]
